# Notes and News

**Published:** 1898-09-10

**Authors:** 


### NOTES AND NEWS.

(Collected and Communicated.)

The German Society of Alienist Physicians will meet at Bonn on the 16th inst.

Madame Simonnet, a patient at the Salpetrière, died recently, aged 105 years.

New Army regulations just passed secure to soldiers any surgical appliance they may require to use through disablement or injury whilst in the service.

Professor Virchow, who is to deliver the second Huxley lecture at Charing Cross Hospital Medical School on October 3rd, will afterwards be entertained at a dinner, under the presidency of Lord Lister.

The typhoid epidemic at Belfast shows a diminution. The cause of the epidemic is thought to have been pollution of the sobsoil and imperfect removal of refuse matters.

The Queen-Mother of the Netherlands is to receive an offering of 300,000 florins as a testimony of the love and esteem felt for her by the people of Holland. The Queen intends to devote the money to philanthropic works, and proposes to found a hospital for poor consumptives of Dutch extraction, and also to assisting the Dutch poor in the East Indies.

It is with great satisfaction that we hear that the Prince of Wales was able last Saturday to put his feet to the ground, and to take a few short steps. This is within seven weeks of the accident, and is very encouraging. There will, however, have to be a good deal of waiting yet before any useful movement can be expected in a joint which has been so seriously injured.

The report of Dr. Harris, medical officer of health for Islington, will not surprise consumers of milk in London. He says that however undiluted and unadulterated milk may be when it arrives from the country, it seldom reaches the public in the same condition, for the Somerset House standard leaves the addition of 6 per cent. of water possible before an offender can be convicted.

The fruit trees in Waterlow Park are this year producing very good crops, which the London County Council intend to present to the various metropolitan hospitals.

A harrowing collection of facts relating to so-called "table delicacies" has been filling the columns of the press. There can be no doubt that everyone runs a considerable risk of poisoning in the consumption of his daily food, and the vigilance of those employed in the inspection of supplies cannot be too highly prized.

A branch institute for the administration of the hot-air treatment by the Tallerman method has recently been opened in Manchester. The influence of this treatment upon painful and stiff joints is in some cases very striking, but the effects upon the system at large which follow upon its merely local application are even more interesting.

Amongst those who received the honorary degree of science at the International Congress of Zoology at Cambridge were: Henry P. Bowditch, professor of physiology, Harvard University; Camillo Golgi, professor of general pathology, Pavia University; Hugo Kronecker, professor of physiology, Berne University; and W. Kuhne, professor of physiology, Heidelberg University.

A new hospital for infectious diseases has been provided for the Lanarkshire Middle Ward, and is situated near Motherwell. The institution is planned to receive 100 patients. The blocks are placed at a distance one from the other, and contain four main pavilions, each of two wards, and others for isolation and observation wards and administration purposes. The buildings are connected by a system of telephones and a complete system of subways. The cost of the building will be about £37,000, or £370 per bed.

The recently published report of the Commissioners on Lunacy shows that they are far from satisfied with the way affairs have been managed at Colney Hatch. They regard the asylum as understaffed. They look upon this fact as partly the cause of the unusually large number of charges of harsh treatment against the male attendants. The arrangements for protection against fire receive especially severe comments at the hands of the Commissioners, and the quality of the food is unhesitatingly condemned, whilst the verdict on the clothing of the patients is little less lenient. The report was made in the spring, and, therefore, the asylum authorities have had ample time to effect the necessary reforms.

Although the season at Nauheim does not really end until October 1, by the time these words are in print it will be practically deserted by the English, and everyone will be enjoying the *nach-kur* on the Black Forest and Swiss hills. In a late number of The Hospital we referred to the professorial honours lately conferred on Dr. Schott by the Grand Duke of Hesse, and now we have to chronicle that he has just received the Order of the Iron Cross from the Emperor of Austria. It is well known that the Empress of Austria has for several weeks been under Professor Schott's treatment, and she left Nauheim at the end of August with her health restored. It is always pleasing to record the reception of honours by medical men, especially when they indicate appreciation of medical skill.

